# Variability in metabolites produced by *Talaromyces pinophilus* SPJ22 cultured on different substrates

**DOI:** 10.1186/s40694-022-00145-8

**Published:** 2022-10-28

**Authors:** Oluwasola Abayomi Adelusi, Sefater Gbashi, Janet Adeyinka Adebiyi, Rhulani Makhuvele, Oluwafemi Ayodeji Adebo, Adeola Oluwakemi Aasa, Sarem Targuma, Glory Kah, Patrick Berka Njobeh

**Affiliations:** 1 Department of Biotechnology and Food Technology, Faculty of Science, University of Johannesburg, Doornfontein Campus, P.O.BOX 17011, Gauteng, South Africa; 2 Department of Chemistry, Faculty of Science, University of Johannesburg, Doornfontein Campus, P.O.BOX 17011, Gauteng, South Africa

**Keywords:** Metabolites, Fungi, *Talaromyces pinophilus*, GC-HRTOF-MS, Substrates

## Abstract

**Background:**

Several metabolites released by fungal species are an essential source of biologically active natural substances. Gas chromatography high resolution time-of-flight mass spectrometry (GC-HRTOF-MS) is one of the techniques used in profiling the metabolites produced by microorganisms, including *Talaromyces pinophilus*. However, there is limited information regarding differential substrates’ impacts on this fungal strain’s metabolite profiling. This study examined the metabolite profile of *T. pinophilus* strain SPJ22 cultured on three different media, including solid czapek yeast extract agar (CYA), malt extract agar (MEA) and potato dextrose agar (PDA) using GC-HRTOF-MS. The mycelia including the media were plugged and dissolved in 5 different organic solvents with varying polarities viz.: acetonitrile, dichloromethane, hexane, 80% methanol and water, and extracts analysed on GC-HRTOF-MS.

**Results:**

The study revealed the presence of different classes of metabolites, such as fatty acids (2.13%), amides (4.26%), alkanes (34.04%), furan (2.13%), ketones (4.26%), alcohols (14.89%), aromatic compounds (6.38%), and other miscellaneous compounds (17.02%). Significant metabolites such as acetic acid, 9-octadecenamide, undecanoic acid methyl ester, hydrazine, hexadecane, nonadecane, eicosane, and other compounds reported in this study have been widely documented to have plant growth promoting, antimicrobial, anti-inflammatory, antioxidant, and biofuel properties. Furthermore, *T. pinophilus* grown on PDA and MEA produced more than twice as many compounds as that grown on CYA.

**Conclusion:**

Thus, our result showed that the production of essential metabolites from *T. pinophilus* is substrate dependent, with many of these metabolites known to have beneficial characteristics, and as such, this organism can be utilised as a sustainable and natural source for these useful organic molecules.

**Supplementary Information:**

The online version contains supplementary material available at 10.1186/s40694-022-00145-8.

## Background

Metabolites are the intermediates products of cellular metabolisms catalysed by different enzymes. The biosynthesis of metabolites by microorganisms has recently gained attention [1]. These metabolites, which include a wide range of antibiotics, antitumor agents, and several therapeutic compounds, are produced naturally in nature as byproducts of microorganisms’ primary or secondary metabolism. Fungi, like other microbes, produce an array of significant metabolites with biotechnological applications [1, 2]. Approximately 250 volatile metabolites from fungi have previously been discovered as intermediates or final products of several metabolic pathways [3]. However, this number has recently been updated to 479 [1]. It has been established that the metabolite profiling of certain fungal species or strain varies depending on the substrate, species, incubation time, and interactions between certain environmental factors [2, 4].

*Talaromyces pinophilus*, a fungus from the genus *Talaromyces* and the family Trichocomaceae is known for producing some essential bioactive metabolites, including terpenoids, alkaloids, polyketides, tetraene, esters, lactones, and furanosteroids [5–7]. This fungal species has been widely employed as effective cellulose and waste degrading agent [8, 9], a renewable source of natural colourants [10], and a stimulator of phytoremediation efficacy [11]. Furthermore, *T. pinophilus* exhibited plant development-promoting properties on Waito-C rice [12] and chickpea [13], as well as mycoparasitic activity against *Botrytis cinerea* [14] and *Rhizoctonia solani* [15]. Interestingly, 3-O-methylfunicone, a prominent metabolite of *T. pinophilus* strain F36CF has been shown to have insecticidal activity against aphids [7] and antiviral effect on Bovine Herpesvirus 1 infection [16], while 2-hydroxyradiclonic acid, a methanolic extract of *T. pinophilus* strain AF-02 obtained from a Chinese green onion, demonstrated potent antibacterial activity against *Escherichia coli* [17]. However, the metabolites produced by *T. pinophilus* are still poorly understood and have not been explored in depth due to lack of comprehensive genetic data [6] and effective metabolomic analytical approach.

Different analytical techniques generally employed in metabolites studies include solid-phase microextraction (SPME), high-performance liquid chromatography (HPLC), nuclear magnetic resonance (NMR), and gas chromatography-mass spectrometry (GC-MS) [18–21]. Thus, the selection of a particular approach is informed by certain factors, including sample matrix, sample quantity, the concentration, and properties of the metabolites [22]. GC-MS, the preferred technique for low polarity, volatile, and semi-volatile compounds [21], is one of the first techniques used in several disciplines for metabolite/metabolomics profiling [23]. Furthermore, GC coupled to high-resolution time-of-flight mass spectrometry (HRTOF-MS) is one of the recent innovations and robust metabolomic technologies, with improved mass resolutions and data collection rates [24, 25]. As shown in previous studies, GC-HRTOF-MS is a potent analytical technique that can effectively screen various metabolites in microorganisms with high sensitivity and excellent results [26–28].

Due to the involvement of *T. pinophilus* in various types of associations with plants and related pathogens and pests, coupled with their biological applications, particularly in crop protection and growth enhancement, the identification of bioactive metabolites with these important biological activities can aid in the development of novel biofungicides, biobactericides, biopesticides, biofertilisers, and biofuel, especially when they are primarily or only found in *T. pinophilus* [21]. This study aimed to examine the diversity in the essential metabolites produced by *T. pinophilus* from South African dairy feed in different media using GC-HRTOF-MS as well as classify the extracted compounds according to their chemical nature.

## Results

### Identification of ***T. pinophilus*** strain SPJ22

**Fig. 1:**
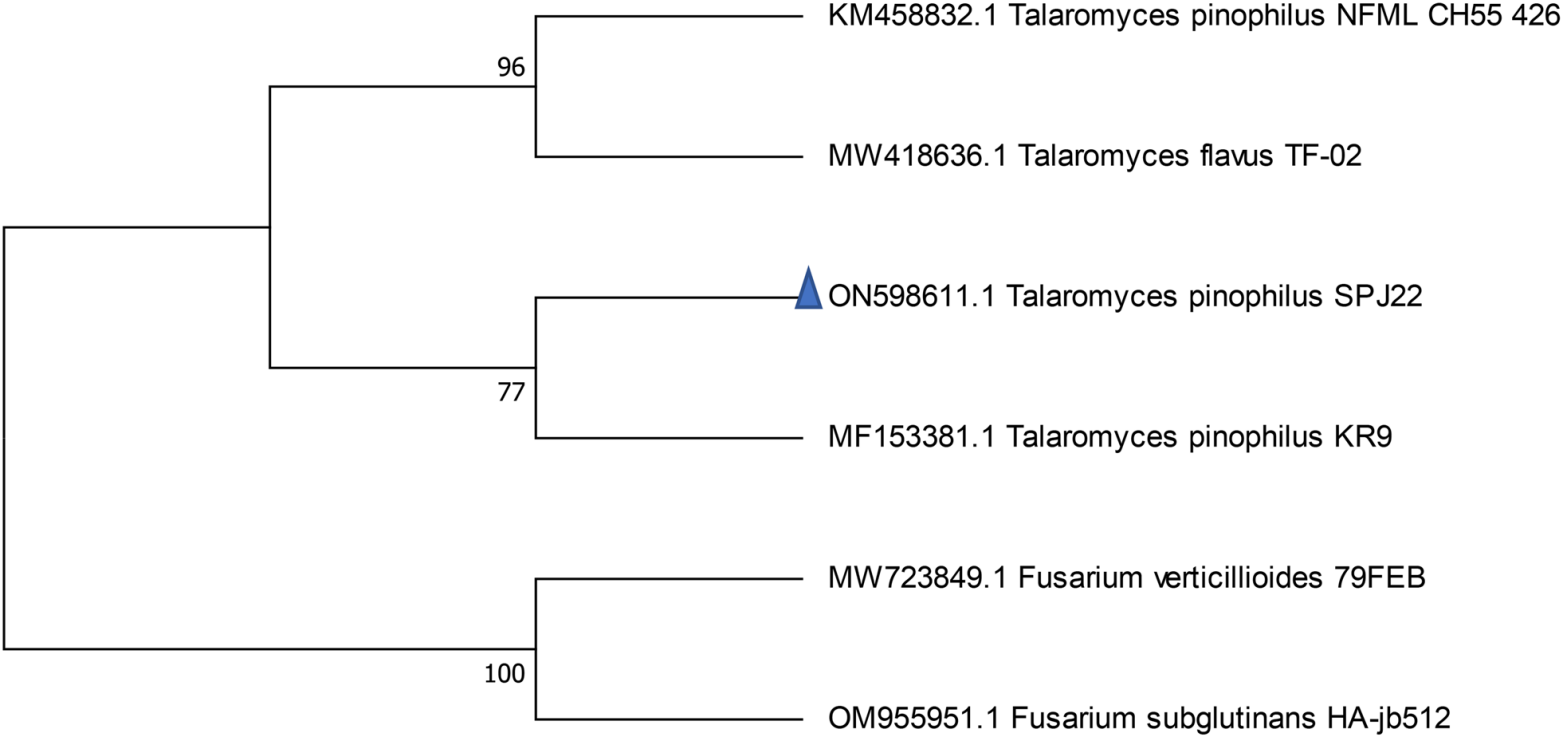
Molecular phylogeny of *T. pinophilus* strain SPJ22 recovered from South African dairy cattle feeds and feedstuffs with DNA sequences for reference strains of *T. pinophilus*. Bootstrap values (as percentages) are shown at internal nodes

*T. pinophilus* strain SPJ22 was identified using both morphological features and molecular approaches. Its conidia germinated on PDA, CYA, and MEA within 5 days at 27 ^o^C. Colour variations were observed in the three media. To confirm the relationship between the *Talaromyces* spp. and *T. pinophilus* strain SPJ22, a phylogenetic tree based on the ITS gene sequence of SPJ22 (ON598611) and other species of *Talaromyces* was constructed (Fig. 1). SPJ22 clearly clustered with *T. pinophilus* strain KR9 with 77% identity.

### **Identification of different metabolites produced by*T. pinophilus*strain SPJ22**

The metabolites of *T. pinophilus* grown on three different substrates (PDA, CYA, and MEA) were analysed using GC-HRTOF-MS. According to our knowledge, this is the first study to investigate the variability in the metabolites produced by *T. pinophilus* on different substrates. The metabolites analysis of the SPJ22 strain revealed the presence of 47 known metabolites (Table 1). The primary metabolites found in the PDA medium include 2-undecen-4-ol, ergosta-5,7,9 (11), 22-tetraen-3β-ol, 1-Iodo-2-methylundecane, 3,3-dimethylpentane, nanonamide, eicosanoic acid, tridecanoic acid methyl ester, 2-hydroxyethyl ester, dibuty phthalate and 2-propynenitrile, 3-fluoro. Hydrazine and Benzeneethanamine, 2-fluoro- beta., 3, 4-trihydroxy-N-isopropyl were the 2 compounds only found in CYA, while 2,2,3,3,5,6,6-heptamethylheptane, 9-octadecenamide, 3-methyl-1,4-diazabicyclo [4.3.0] nonan-2,5-dione,N-acetyl, hexahydropyrrolo[1,2-a]pyrazine-1,4-dione and 3,3-di (trifluoromethyl) diazirine were the primary metabolites found only in MEA (Table 1).

**Table 1:** Identified metabolites produced by *T. phinophilus* on PDA, MEA, and CYA.

Metabolites	RT (min)	MF	m/z	Media	Solvent
Fatty acid (1)					
Acetic acid	2.73	C_2_H_4_O_3_	41.08	C, P	A, D, H, E
Alcohols (7)					
1- Octanol,2,2,3,3,4,4,5,5,6,6,7,7,78,8,8-pentadecafluoro	3.73	C_8_H_3_F_15_O	226.25	C, P	H, E
1,2-Ethanediol	2.70	C_2_H_6_O_2_	33.08	M	W, H, E
2-Chloroethanol	2.78	C_2_H_5_ClO	84.96	C, P	A, D, H
2-Undecen-4-ol		C_11_H_22_O	169.19	P	D, H
Ergosta-5,7,9 (11), 22-tetraen-3β-ol	27.12	C_28_H_42_O	394.325	P	D
Ethanol,2,2-dichloro	2.60	C_2_H_4_Cl_2_O	49	C, M	A, D, H
Methanol	2.84	CH_4_O	31.06	C, M, P	A, D, E, H, W
Alkanes (16)					
1-Iodo-2-methylundecane	9.83	C_12_H_25_I	268.98	P	H
2,2,3,3,5,6,6-Heptamethylheptane	9.58	C_14_H_30_	169.19	M	D
3,3-dimethylpentane	6.83	C7H_16_	101.04	P	H
Eicosane	10.76	C_20_H_42_	270.24	M, P	D, H
Heneicosane	17.92	C_21_H_44_	248.56	M, P	D, H
Heptadecane	11.41	C_17_H_36_	235.11	M, P	D, H
Heptadecane,2,6,10,15-tetramethyl	13.33	C_21_H_44_	292.70	M, P	D, H
Hexacosane	18.65	C_26_H_54_	415.04	P	H
Hexadecane	11.08	C_16_H_34_	226.43	M, P	D, H
Nonadecane	14.98	C_19_H_40_	268.44	M, P	D, H
Octadecane	13.48	C_18_H_38_	250.68	M, P	D, H
Pentacosane	20.78	C_25_H_52_	324.98	P	D
Pentadecane	10.71	C_15_H_32_	219.06	M, P	D, H
Tetradecane	7.39	C_14_H_30_	198.24	P	H
Triacontane	19.42	C_30_H_62_	267.86	M, P	D, H
Tris(trifluoromethyl)bromomethane	9.16	C4BrF9	219.11	P	W
Amides (2)					
9-Octadecenamide	21.12	C_18_H_35_NO	281.27	M	E
Nonanamide	21.10	C_9_H_19_NO	156.14	P	D
Aromatic compounds (3)					
2,4-Di-tert-butylphenol	11.31	C_14_H_22_O	206.17	M, P	D, H
Benzeneethanamine, 2-fluoro-. beta., 3, 4-trihydroxy-N-isopropyl	21.18	C_11_H_16_FNO_3_	154.12	C	D
Phosphinothioic fluoride, (1,1-dimethylethyl) pentaflurophenyl	13.76	C_10_H_9_F_6_P_5_	227.16	P	H
Esters (7)					
Benzenepropanoic acid, 3,5-bis (1,1-dimethylethyl)-4-hydroxy-, methyl ester	17.12	C_18_H_28_O_3_	292.20	P	D, H
Dibuty phthalate	17.43	C_16_H_22_O_4_	150.03	P	A
Eicosanoic acid, 2-hydroxyethyl ester	21.70	C_22_H_44_O_3_	401.98	P	D
Pentadecanoic acid, 14-methyl-, methyl ester	16.91	C_17_H_34_O_2_	270.26	P	A
Sulfurous acid, 2-pentyl pentyl ester	10.69	C_10_H_22_O_3_S	223.56	P	H
Tridecanoic acid methyl ester	17.43	C_14_H_28_O_2_	227.45	M, P	A, D, E
Undecanoic acid methyl ester	18.25	C_12_H_24_O_2_	200.17	P	A, D, H
Furan (1)					
5-Methyl-2-(2-methyl-2-tetrahydrofuryl) tetrahydrofuran	11.33	C_10_H_18_O_2_	169.20	M, P	D, H
Ketones (2)					
3-Methyl-1,4-diazabicyclo [4.3.0] nonan-2,5-dione, N -acetyl	14.89	C_10_H_14_N2O_3_	219.15	M	A
Hexahydropyrrolo[1,2-a] pyrazine-1,4-dione	15.32	C_7_H_10_N_2_O_2_	151.05	M	A
Others (miscellans compounds) (8)					
1- Dimethyl (prop-2-enyl) silyloxypentane	9.61	C_10_H_22_OSi	157.10	P	H
1,1,1,2,3,3,3-Heptafluoro-2-methoxypropane	10.26	C_4_H_3_F_7_O	224.97	M	A, H, W
2-Propynenitrile, 3-fluoro	11.02	C_3_FN	69.09	P	H
3,3-Di(trifluoromethyl)diazirine	15.26	C_3_F_6_N_2_	131.15	M	E
Hydrazine	2.65	H_4_N_2_	32.06	C	A, H
Phosphine, Tris (trifluoromethyl)	12.04	C_3_F_9_P	224.78	C, M, P	A, D, E, H, W
Silane	2.61	H_4_Si	31.99	P	H
3,5-Cyclo-6,8(14), 22-ergostratriene	27.38	C_28_H_42_	378.33	M	D

*PDA* potato dextrose agar,* MEA* malt extract agar,* CYA* czapek yeast extract agar,* RT* Retention time,* MF* Molecular formula,* m/z* mass-to-charge ratio,* M * Malt extract agar,* C*  Czapek yeast extract agar,* P*  Potato dextrose agar,* A* Acetonitrile,* D* Dichloromethane,* H*  Hexane,* E* 80% Methanol,* W*  water

**Fig. 2:**
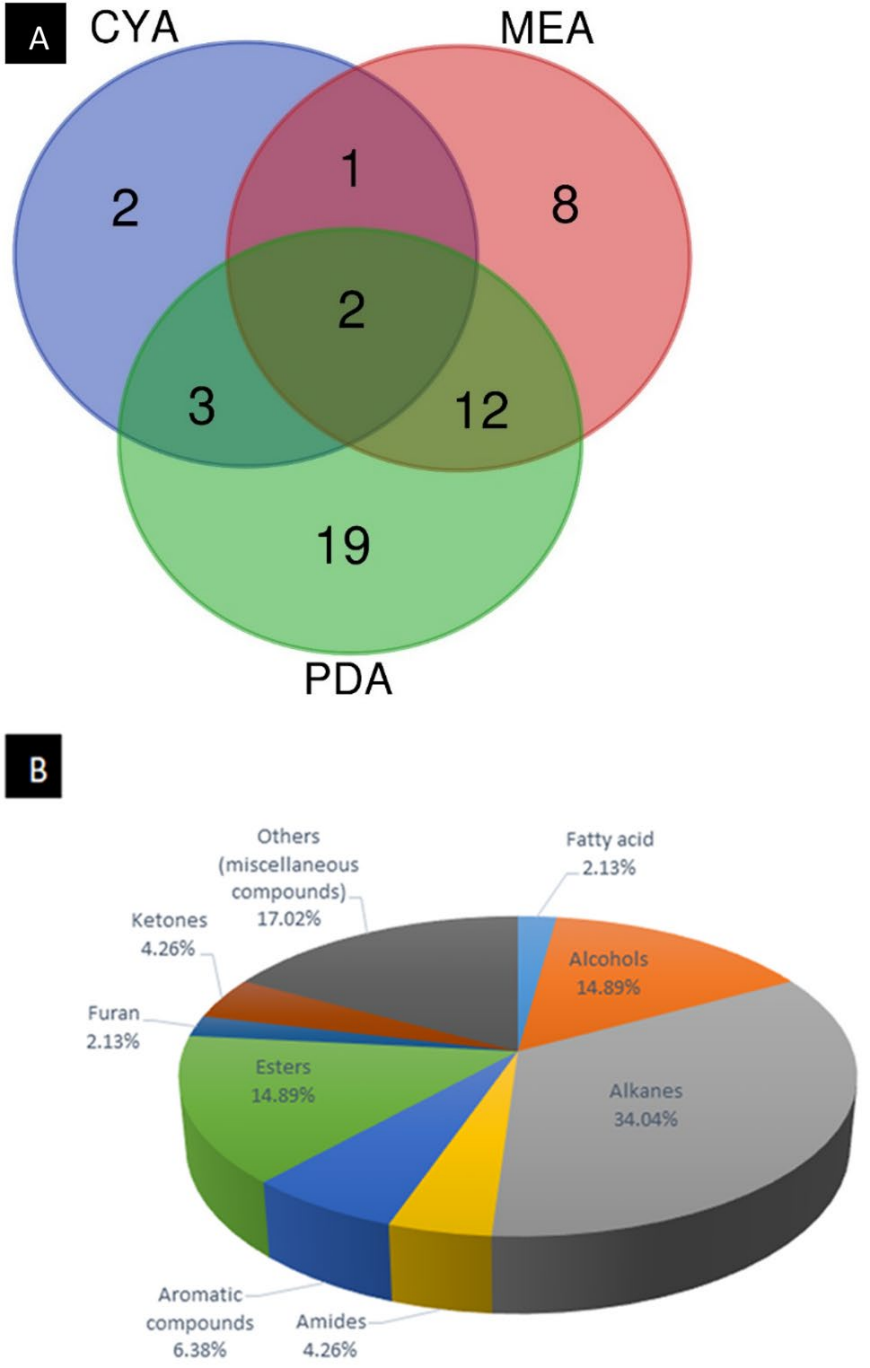
**A** Venn diagram showing the number of metabolites produced by the *T. pinophilus* on the three cultivation media (CYA, PDA, and MEA), and **B** Pie chart showing percentage distribution of the metabolites produced by *T. pinophilus*

PDA exhibited greater product diversity than CYA, with 31 substances distinct from those produced on CYA, and 12 in common with those of MEA, showing the organism’s broad capacity to produce different compounds on diverse substrates (Fig. 2A). Most compounds produced on CYA were also produced on either MEA or PDA. The identified metabolites in the different intersections of the substrate are shown in Additional file 1.

The compounds detected in this study were further grouped according to their chemical nature, including alkanes (34.04%), alcohols (14.89%), ketones (4.26%), esters (14.89%), aromatic compounds (6.38%), amides (4.26%), furan (2.13%), fatty acid (2.13%), and other miscellaneous compounds (17.02%) (Fig. 2B). Surprisingly, all 7 esters produced by the *T. pinophilus* strain SPJ22 were detected in PDA and MEA cultures, with 6 of these metabolites only found in PDA. Alkanes, the highest occurring group of compounds produced by *T. pinophilus* in this study, were only found in MEA and PDA cultures, while the only 2 ketones identified in this work were only observed in MEA culture.

## Discussion

Several authors have reported the significant impact of substrate composition on the type and frequency of metabolites released by fungal isolates [29–31]. In this study, *T. pinophilus* grown on PDA and MEA produced more than twice as many compounds than *T. pinophilus* grown on CYA. This same trend was also observed in a previous work by Mallette et al. [32], which could be attributed to differences in substrates, which in turn could alter microbial activities. This is unsurprising given the disparities in initial substrate complexity. It has also been established that *Penicillium aurantiogriseum* produced the highest number of alcohol when inoculated on oat grain, and equally released more terpenes when grown on Norkrans and Czapek agars [33]. In another study conducted by Larsen and Frisvad [34] to characterise the secondary metabolites from 47 *Penicillium* taxa using Fourier transform infrared spectroscopy (FTIR) and GC-MS, the authors confirmed that metabolites profiling of the fungal species vary with the substrates as the *Penicillium* isolates grown on yeast extract sucrose (YES) agar produced the highest amount of metabolites, while the same isolates produced a much smaller amount of metabolites when cultured on malt extract agar (MEA), with the isolates inoculated on CYA producing the least amount of compounds.

Furthermore, a research performed to evaluate the impacts of substrate and fungal species on the production of secondary metabolites by two *Trichoderma* spp. *i.e., T. viride* (T60) and *T. pseudokoningii* showed that the metabolites released by the 2 *Trichoderma* spp. were dependent on both substrate type and the *Trichoderma* isolate [35]. This was in concordance with Mäki et al. [4] who investigated the influence of different wood substrates (pine wood and spruce wood substrates) on the production of essential metabolites by 3 wood-decaying fungal isolates, *Phlebia radiata*, *Fomitopsis pinicola* and *Trichaptum abietinum*. Their findings revealed that substrate quality might enhance the release of metabolites from fungal species as the concentration of the significant metabolites released on pine wood substrate were higher than on spruce wood substrate.

Some of the metabolites produced by *T. pinophilus* strain SPJ22 in this work had previously been attributed to *Talaromyces* by Zhai et al. [5] and they are widely documented to have plant growth promoting, antioxidant, antimicrobial, anti-inflammatory, and biofuel properties [26, 36, 37]. For instance, it has been shown that acetic acid increases grapevine tolerance to NaHCO_3_ stress by raising salicylic acid, the endogenous growth regulator of phenolic nature [38]. Pentadecane and 1-iodo–2-methylundecane obtained via co-culturing of *Trichoderma longibrachiatum* with *Macrophomina phaseolina*, *Rhizoctonia solani*, and *Magnaporthe grisea* in rice clearly displayed mycoparasitic activity against *R. solani, Pythium* sp., *M. grisea, Fusarium oxysporum*, *M. phaseolina*, and *Cyrtomium falcatum* with a more significant effect demonstrated on *R. solani* [39]. Furthermore, undecanoic acid methyl ester, another important metabolite released by *T*. *pinophilus* SPJ22 has been found to possess antioxidant and antimicrobial properties [40, 41].

Hydrazine is a precursor to a variety of pharmaceuticals and pesticides. This substance has also been found to have antifungal effects against a number of fungal species. For instance, Dascalu et al. [42] investigated the antifungal properties of hydrazine compound and its derivatives against 12 fungal species viz. *Penicillium ochrochloron*, *Cladosporium cladosporioides*, *Paecilomyces variotii*, *Alternaria alternata*, *Aspergillus oryzae*, *Sclerotinia sclerotiorum*, *Botrytis cinerea*, *Fusarium solani*, *Candida tropicalis*, *Geotrichum candidum, Candida pseudotropicalis*, and *Candida krusei*, with good antifungal activities, particularly against *P. variotii* and *F. solani.* Moreso, Zakaria et al. [43] showed that hexadecane has a strong inhibitory effect on *Pseudomonas aeruginosa*, demonstrating antioxidant and antibacterial properties. Eicosane has been confirmed to have activity on clinical and food borne pathogens [44, 45], whereas 9-octadecenamide was demonstrated to have antibacterial and anti‐inflammatory properties [37, 46].

Biofuel (ethanol and biodiesel) is a potential liquid fuel currently utilised as an alternative fuel for transportation [47]. Fatty acid methyl esters (FAMEs), which make up biodiesel, are now derived from microbial sources such as fungi, bacteria, and algae. Mallette et al. [32] identified nonadecane as one of the most important biofuel compounds produced by *Ascocoryne sarcoides* (NRRL 50,072). Finally, it is important to mention that most of the alkanes released by *T. pinophilus* strain SPJ22 has been reported to be potential bio-renewable fuel/mycodiesel [48].

The production of beneficial biotechnological metabolites by the *T. pinophilus* isolate in this study can be linked to the putative biosynthetic gene cluster previously reported in some fungal isolates, including the genera *Talaromyces* [6, 49]. A comparative genomic analysis of *T. pinophilus* 1–95 revealed that the fungal strain contained 68 metabolism gene clusters containing 401 putative genes, including Type1 polyketide synthase genes and nonribosomal peptide synthase genes [6]. The authors found that *T. pinophilus* 1–95 contains more secondary metabolites than other related filamentous fungi, promoting the cultivation of *T. pinophilus* for the high synthesis of beneficial metabolites. In addition, Ahmed [49] complete genome sequence of *T. stipitatus* and the advancement in bioinformatics tools have facilitated the discovery of the fungus’s biosynthetic potential, with the identification of a putative biosynthetic gene cluster (BGC) of the polyesters encoding a highly reducing polyketide synthase (HR-PKS) and nonreducing polyketide synthase (NR-PKS). According to Le Govic et al. [50], these genes are responsible for forming several metabolites in bacterial and fungal species.

## Limitations

The main limitation of this study was the agar plug extraction method, as other compounds from the fungus and media could have been extracted along with the metabolites. Another limitation is the inability to identify the specific biosynthetic gene clusters involved in producing the essential metabolites in *T. pinophilus* SPJ22.

## Conclusion

Several metabolites of *T. pinophilus* were identified in this study using three different substrates (i.e., PDA, CYA, and MEA). Accordingly, the type of metabolites and frequency of their production show conclusively that metabolites production by fungi, particularly *T. pinophilus*, are substrate dependent. The application and potential use of the important metabolites released by this fungal strain in the medical, industrial, and agricultural sectors is feasible. These substances can be employed in cost-effective biological processes to boost agricultural productivity, as some of these compounds have been widely documented to have biofertilizer, antimicrobial, mycotoxin biocontrol, and biofuel-producing properties. Further research into the separation, quantification and application of the metabolites found in this study is still needed, particularly for those important bioactive metabolites obtained in all three media. These findings contributed to a better understanding of metabolites produced by *T. pinophilus* SPJ22 (ON598611) in various substrates.

## Materials and methods

### Isolation and identification of ***T. pinophilus***

The *T. pinophilus* SPJ22 strain used in this study was recovered from dairy cattle feeds in South Africa. The fungus strain was identified macroscopically and microscopically using the identification method of [51] and confirmed by molecular means. Deoxyribonucleic acid (DNA) was extracted from a 7-day-old *T. pinophilus* inoculated on PDA using the Fungal/Bacterial DNA extraction kit (Zymo Research Corporation, Southern California, USA) as directed by the manufacturer. Then, the isolated DNA was amplified at the ITS region using the primers ITS-1; 5’- TCC GTA GGT GAA CCT GCG G − 3’ (forward) and ITS-4; 5’- TCC TCC GCT TAT GC-3’ (reverse), designed by White et al. [52]. Each reaction contained 12.5 µL of Red taq ready mix (Sigma- Aldrich, Germany), 0.3 µL of each primer (ITS-1 and ITS-4), 0.8 µL of DNA sample, 0.5 µL of dimethyl sulfoxide (DMSO), and 9.6 µL of ddH_2_O to bring the total volume to 24 µL. A negative control comprising all reagents besides the DNA was also prepared.

A ProFlex 32-well PCR System (ThermoFisher Scientific, Singapore) was used to perform the PCR reaction with initial DNA denaturation at 95 ^o^C for 2 min, 35 cycles of denaturation at 95 ^o^C for 30 s, an annealing step at 50 ^o^C for 30 s and primer extension at 72 ^o^C for 1 min. This was followed by a final elongation period of 10 min at 72 ^o^C holding for 4 ^o^C until samples were retrieved. After that, PCR products were purified with a DNA ZR-96 sequencing clean-up kit (Applied Biosystems, Foster City, CA, USA) to remove residual primers. Purified PCR products were sequenced in both directions (forward and reverse) at the African Centre for DNA Barcoding (ACDB), University of Johannesburg, South Africa, using an ABI 3130 x l Genetic Analyzer (ThermoFisher Scientific, Tokyo, Japan). The DNA sequence was analysed using BLAST (http://blast.ncbi.nlm.nih.gov/Blast.cgi) to obtain the species name.

The 18 S rRNA gene sequences from the GenBank database were used as the baseline for phylogenetic analysis and multiple data alignments using ClustalW of the EMBL-EBI website (https://www.ebi.ac.uk/Tools/msa/clustalo). Afterwards, a phylogenetic tree was constructed with the help of MEGA 7.0 [53] by measuring distances and clustering using the Maximum Likelihood strategy of [54]. The parameter chosen for the phylogenetic tree construction was Bootstrap values based on 1000 replications [55], and all branches with less than 50% site coverage were collapsed. *Fusarium verticillioides* and *Fusarium subglutinans* were selected as the out-group species. The phylogenetic tree constructed was utilised to evaluate the evolutionary relationship between the isolated *T. talaromyces* strain SPJ22 from this study and its Gen Bank relatives. Sequence was then deposited in a GenBank under the accession number ON598611.

### Extraction of metabolites

Pure *T. pinophilus* strain SPJ22 was sub-cultured unto petri dishes containing solidified PDA, MEA, and CYA and incubated at 27 ^o^C in darkness for three weeks. Five different solvents (acetonitrile, dichloromethane, hexane, 80% methanol and water) were further utilised to extract the metabolites from the cultured fungus. For the extraction of metabolites, 5 g of the isolate, including the medium, was plugged into a centrifuge tube containing 10 mL of each solvent. The content was agitated for 1 h using a Labcon shaker (Labcon, California, USA) and thereafter, filtered using a Whatman no. 4 filter paper (Merck, Johannesburg, SA). A freeze-drier was used to concentrate the filtrate and dried extract reconstituted using 5 mL of LC-MS grade methanol. Approximately 1.5 mL of the extract was filter-sterilised and transferred into dark amber vials for GC-HRTOF-MS analysis. For each sample, extraction of metabolites was done in triplicates.

### GC-HRTOF-MS analysis

The samples were analysed with a LECO Pegasus mass spectrometer (LECO Corporation, St Joseph, MI, USA) equipped with a modified Agilent 7890 A Gas Chromatograph containing an oven and a split/splitless inlet (Agilent Technologies, Inc., Wilmington, DE, USA). The column utilised was a Rxi-5 SilMS (29.5 m × 0.25 mm × 0.25 μm) (Restek, Bellefonte, PA, USA). The carrier gas used was helium pumped at a constant flow rate of 1 mL/min with an inlet temperature of 250  °C. An initial oven temperature of 40 °C was set and maintained for 0.5 min and slowly ramped at a rate of 10  °C/min to 250  °C and held for about 0.5 min. The mass spectrometer was configured as follows: source temperature at 250 °C; electron ionisation at − 70 eV; transfer line temperature at 250 °C; stored mass range at 45–600; acquisition rate at 10 spectra/s for GC-HRTOF-MS; and detector offset voltage at 300 V.

### Data processing and statistical analysis

A ChromaTOF software (LECO, USA) was used for matched filtering, peak identification, and retention time alignment. After that, each compound was identified by comparison with mass spectral databases (NIST, Adams, and EO libraries), and a semi-quantification of each molecule was established using peak regions and relative concentration expressed in percentage.

## Supplementary Information

**Additional file 1**. Identified metabolites in the different intersections of the Venn diagram.

## Data Availability

The raw datasets obtained in this work are available upon request from the corresponding authors.

## Abbreviations

GC-HRTOF-MS: Gas chromatography high resolution time-of-flight mass spectrometry
CYA: Czapek yeast extract agar
MEA: Malt extract agar
PDA: Potato dextrose agar
DMSO: Dimethyl sulfoxide
LC-MS: Liquid chromatography–mass spectrometry
