# Supplementary material for: Variability in metabolites produced by Talaromyces pinophilus SPJ22 cultured on different substrates
Source: Fungal Biol Biotechnol. 2022 Oct 28;9:15. doi: 10.1186/s40694-022-00145-8 (PMC9617411; doi:10.1186/s40694-022-00145-8)
Supplement: Supplementary file 1 — Additional file 1. Identified metabolites in the different intersections of the Venn diagram. [file 40694_2022_145_MOESM1_ESM.docx]

**Additional file 1**: Identified metabolites in the different intersections of the Venn diagram.

| **Names of substrate** | **Number of metabolites** | **Names of metabolites** |
| --- | --- | --- |
| CYA MEA PDA | 2 | Phosphine, tris(trifluoromethyl) and Methanol. |
| CYA MEA | 1 | Ethanol, 2,2-dichloro. |
| CYA PDA | 3 | Acetic acid, 2-Chloroethanol and 1-Octanol, 2,2,3,3,4,4,5,5,6,6,7,7,8,8,8-pentadecafluoro |
| MEA PDA | 12 | Tridecanoic acid methyl ester, 2,4-Di-tert-butylphenol, Triacontane, Eicosane, Hexadecane, Heneicosane, 5-Methyl-2-(2-methyl-2-tetrahydrofuryl) tetrahydrofuran, Nonadecane, Pentadecane, Heptadecane, Heptadecane, 2,6,10,15-tetramethyl and Octadecane |
| CYA | 2 | Hydrazine and Benzeneethanamine, 2-fluoro-. beta., 3, 4-trihydroxy-N-isopropyl. |
| MEA | 8 | Hexahydropyrrolo[1,2-a] pyrazine-1,4-dione, 9-Octadecenamide, 3-Methyl-1,4-diazabicyclo [4.3.0] nonan-2,5-dione, N-acetyl, 1,2-Ethanediol, 2,2,3,3,5,6,6-heptamethyl heptane, 1,1,1,2,3,3,3-Heptafluoro-2-methoxypropane, 3,3-Di(trifluoromethyl)diazirine and 3,5-Cyclo-6,8(14),22-ergostatriene. |
| PDA | 19 | 1-Dimethyl(prop-2-enyl)silyloxypentane, 2-Undecen-4-ol, ergosta-5,7,9 (11), 22-tetraen-3β-ol, Pentadecanoic acid, 14-methyl-,methyl ester, Tris(trifluoromethyl)bromomethane, Undecanoic acid methyl ester, Dibutyl phthalate, Phosphinothioic fluoride, (1,1-dimethylethyl) pentafluorophenyl, Hexacosane, Pentacosane, 1-Iodo-2-methylundecane, Nonanamide, 2-Propynenitrile,3-fluoro, Tetradecane, Sulfurous acid, 2-pentyl pentyl ester, 3,3-dimethyl pentane, Silane, Benzenepropanoic acid, 3,5-bis(1,1-dimethylethyl)-4-hydroxy-, methyl ester and Eicosanoic acid, 2-hydroxyethyl ester |

PDA = potato dextrose agar; MEA = malt extract agar; CYA = czapek yeast extract agar.
